# Facilitating remote and virtual access provision by European research infrastructures – requirements, issues, and recommendations

**DOI:** 10.12688/openreseurope.18023.2

**Published:** 2025-05-16

**Authors:** Michael RAESS, Omran Alhaddad, Johanna Bischof, John Dolan, Ayoub El Ghadraoui, Menattallah Elserafy, Marco Galeotti, Ulla Lächele, Xavier Meyer, Oguz Ozkan, Ivan Rodero, Hannele Savela, John Shepherdson, Vanessa Spadetto, Valentina Tegas, Susanne Vainio, Alen Vodopijevec, Bonnie Wolff-Boenisch, Claudia Alen Amaro, Annika Thies

**Affiliations:** 1 INFRAFRONTIER ERIC, Neuherberg, Bayern, Germany; 2 INSTRUCT-ERIC, Oxford, UK; 3 Bio-Hub, Euro-BioImaging ERIC, Heidelberg, Germany; 4 UNESCO, Paris, France; 5 EMSO-ERIC, Rome, Italy; 6 Deutsches Elektronen-Synchrotron DESY, Hamburg, Germany; 7 European Science Foundation, Strasbourg, Grand Est, France; 8 University of Oulu, Oulu, Northern Ostrobothnia, Finland; 9 INTERACT Nonprofit Association, Malmo, Sweden; 10 CESSDA ERIC, Bergen, Norway; 11 Euro-BioImaging ERIC, Turku, Finland

**Keywords:** Research Infrastructures, Remote Acccess, Virtual Access, Remote Training, Data Sharing

## Abstract

Research infrastructures (RIs) are strategic assets facilitating innovation and knowledge advancement across all scientific disciplines. They provide researchers with advanced tools and resources that go beyond individual or institutional capacities and promote collaboration, community-building and the application of scientific standards. Remote and virtual access to RIs enables scientists to use these essential resources without the necessity of being physically present. The COVID-19 pandemic restrictions where a catalyst for the expansion and further development of remote and virtual access models, particularly in fields where physical access had been the predominant model. The eRImote project collected the experiences gained in different scientific fields through targeted surveys, stakeholder workshops, expert groups discussions, and the analysis of specific use cases, with the aim of identifying good practice and presenting recommendations. This paper provides a definition of remote and virtual access and remote training and explores their implementation across various RIs, highlighting the implications for their operational processes and the dynamics of interaction between RIs and their user communities. It presents the identified advantages, obstacles, and best-practices, alongside strategies and recommendations to navigate and mitigate challenges effectively. Key issues and recommendations are summed up separately for remote access, virtual access, and remote training, complemented by general recommendations for facilitating remote and virtual access to RIs. These relate to budgeting and funding, the balancing of RI access models, the need for regulatory frameworks for sample shipments, collaboration among RIs, impact assessment of remote and virtual access on user interactions, operational efficiency and the environment footprint of RIs, and the adaption of data sharing policies. Stakeholders were broadly invited to give their feedback on the paper’s findings and conclusions, which were integrated into an improved version of this paper.

## The role of remote and virtual access in European research infrastructures

In the ever-evolving landscape of research and scientific discovery, access to cutting-edge research infrastructures (RIs) facilitates innovation and knowledge advancement across all scientific disciplines. Europe features a vibrant ecosystem of national and European-level RIs in various domains of science. The European Strategy Forum on Research Infrastructures (ESFRI) was established in 2002 to develop a coherent and strategic approach to policy-making on RIs in Europe. ESFRI defines RIs as "facilities, resources, and related services that are used by the scientific community to conduct top-level research in their respective fields". These include major scientific equipment (or set of instruments), knowledge-based resources such as collections, archives and scientific data, e-infrastructures like data and computing systems, communication networks, and any other tools essential for excellence in research and innovation" ^1^.

The overarching goal of RIs is to provide researchers with advanced tools and resources that go beyond individual or institutional capacities. By promoting collaboration and open access, RIs are catalysts for accelerating scientific progress and international cooperation. In addition to access provision to scientific platforms, resources, services, expertise and data, an important component of RIs is training to both to users empowering them to use the RI provided resources, but also internally to the RI staff and operators, to facilitate the application of standards and operation procedures. This standardisation and alignment of operational procedures is crucial, particularly for “distributed" RIs, which federate facilities across Europe or globally into a unified infrastructure. RIs also play a vital role as community-building hubs, advocating for their respective community in European and global policy landscapes, and facilitating networking opportunities across institutional and national boundaries.

Remote and virtual access to RIs enables scientists to use these essential resources without physical presence at the installations through web-based platforms, virtualisation technologies, and secure network connections, or by sending samples for analysis. This approach not only simplifies access to state-of-the-art equipment and potentially increases the efficiency of RI utilisation, it also fosters international partnerships and broadens access to valuable resources otherwise challenging to acquire. The definition of remote and virtual access varies based on the service offered and differs across domains and RIs as detailed below.

Even before the COVID-19 pandemic, European RIs had developed various remote and virtual access models ^2^; in some research fields they are the predominant operation model, especially when physical access is impractical due to geographically remote facilities (e.g., astronomical telescopes), when physical access is restricted to RI staff (e.g. high-hygiene or high containment facilities in the biomedical sciences, or where the RI's output is digital and therefore virtually accessible. The pandemic restrictions acted as a catalyst for the expansion and further development of remote and virtual access models, particularly in fields where physical access had been the predominant model. Even though many RI operations often reverted to physical access after the pandemic, remote and virtual access mechanisms were often maintained ^3^. In this way, RIs could increase their preparedness for future emergencies and take advantage of diversified access pathways with a potential for more sustainable (and eco-friendly) operations. As a result, many RIs are now in the process of optimising temporary and provisional solutions from the pandemic into stable and sustainable remote access models.

**Sidebox: The eRImote project**

The eRImote project (European Research Infrastructures - Pathway to Improved Resilience through Digital and Remote Access) ^4^ was developed in response to the COVID-19 pandemic and the need to find new innovative solutions to access RI. The eRImote consortium brings together twelve partners from diverse scientific disciplines - including environmental, life, physical and social sciences - to explore pathways for digital and remote RI access. Through targeted surveys, stakeholder workshops, expert groups discussions, and the analysis of specific use cases ^5^, eRImote aims to identify the benefits, challenges, and barriers for remote and digital RI access.

This paper outlines the project's findings, presenting the identified advantages, obstacles, and best-practices, alongside strategies and recommendations to navigate and mitigate challenges effectively. Stakeholders were broadly invited to give their feedback on the paper’s findings and conclusions, which were integrated into this improved version.

A second major output of the project is the eRImote information platform. It provides RI stakeholders with good practice examples on the various challenges of remote access, talks and video recordings of presentations. It will continue to be available as a repository also beyond the duration of the project. See https://erimote.eu/resources. Many documents and resources referred to in this paper are accessible there ( https://erimote.eu/documents).

## Definitions for remote and virtual access

The European Charter for Access ^6^ provides a comprehensive definition of access to RIs: "The legitimate and authorised physical, remote and virtual admission to, interactions with and use of research infrastructures and to services offered by research infrastructures to users. Such access can be granted, amongst others, to machine time, computing resources, software, data, data-communication services, trust and authentication services, sample preparation, archives, collections, the set-up, execution and dismantling of experiments, education and training, expert support and analytical services."

ESFRI's 2020 White Paper ^7^ elaborates on the differences between physical, virtual, and remote access: *Physical Access* involves “hands-on” access when users physically visit an infrastructure, facility, or equipment. The available services or resources are usually limited, and a competitive process may therefore be required, following a defined procedure and criteria for selection of users. *Remote Access* allows users to utilize RI services or control instrumentation from afar, similarly constrained by resource limitations and competitive entry. *Virtual Access*, provided over communication networks, mainly pertains to data and digital tools, offering broader, more flexible engagement opportunities. As the available services or resources can be used simultaneously by several users, any access restrictions are due to other characteristics such as access to sensitive data. In a recent publication, ESFRI notes that *hybrid* access solutions, combining physical with remote and virtual access are also increasingly applied ^8^.

Remote and virtual access to RIs have the potential to offer benefits in times where technology is advancing, and physical presence is no longer, or less often, required. This access model can play a significant role in removing financial and geographical barriers, thus promoting the democratisation of access to RIs, (e.g. for researchers with care responsibilities or researchers from outside Europe who may face travel restrictions either due to distance or visa issues, see Sidebox Remote access in a global context), contributing to a reduction in the carbon footprint associated with user travel, and most importantly accelerating discovery/progress. Whether virtual and remote access have the potential to improve operational efficiency and the carbon footprint of the overall service delivery is highly dependent on context and implementation. Achieving these benefits requires tailored strategies that consider the unique needs and constraints of each RI. One the other hand, the switch to remote and virtual access requires substantial changes to RI operational models, introduces novel complexities and poses new challenges for RIs, their staff and users. These benefits and disadvantages need to be carefully managed to realise the full potential of the transition.

**Sidebox: Remote access in a global context**

While most European research infrastructures, and many others globally, are openly accessible to all researchers around the world within a user access framework, experience shows that an open access principle is insufficient to ensure equitable access from all world regions ^9^. Remote access can be a highly impactful pathway to increasing equitable access to RI services for researchers that otherwise face barriers to access through e.g. high travel costs and visa challenges. At the same time, it should be noted that many of the challenges and barriers to remote access described in this paper - lack of hands-on training, challenges in sample shipment, lack of sufficient IT infrastructure - are going to impact researchers from low-/middle-income countries (LMIC) more strongly. In-person visits to RI facilities also often play a critical role in building long term collaborations and providing training for researchers from the Global South. Remote access should therefore be seen as one tool within a toolbox of programs that support equitable access for LMIC researchers to European RI resources.

In the following section, we explore the implementation of remote and virtual access and remote training across various RIs, highlighting the implications for their operational processes and the dynamics of interaction between RIs and their user communities. By examining specific examples and strategies employed by RIs to facilitate these modes of access and training, we can better understand the potential benefits, challenges, and best practices that emerge from this digital transformation. This analysis aims to provide insights into how RIs can optimise their services to support a broader range of users while maintaining high standards of research excellence and collaboration.

## Remote access

**Figure 1.**
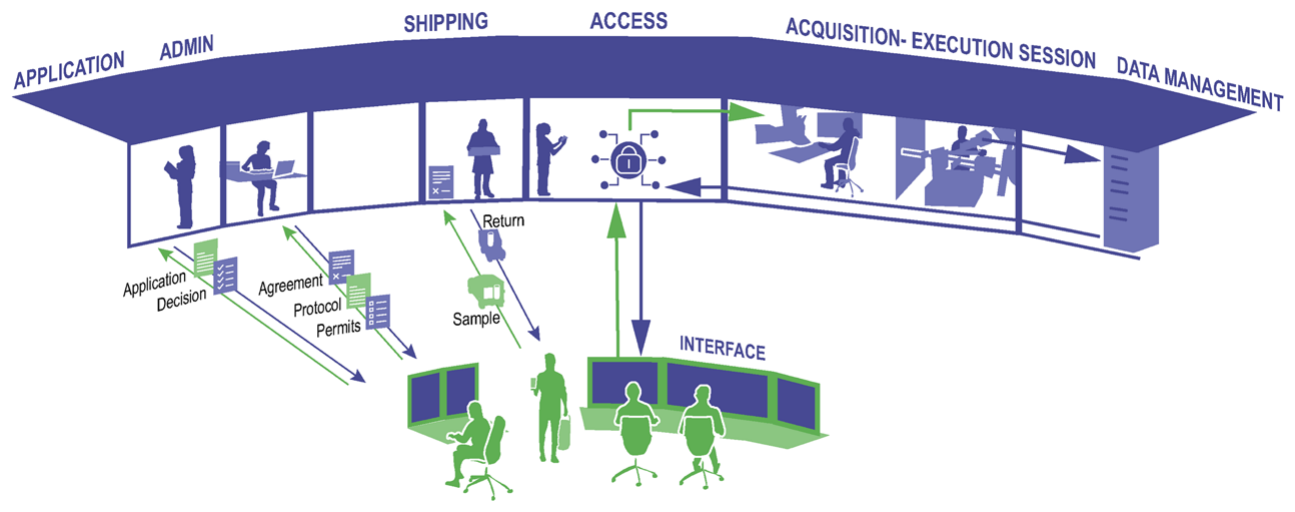
Remote Access: interaction between the user (depicted in green) and the RI staff (blue). Example of the lifecycle of an experiment conducted via remote access, e.g. at a synchrotron or electron microscope, starting from the application of the researcher to use the RI and moving on to sample shipment, the actual measurements/experiments taking place to the management of the resulting data to which the user obtains access.

Remote access to RIs encompasses a range of methodologies tailored to the needs of users and the capabilities of the facilities. In many cases users submit samples which are analysed by the RI, eliminating the need for direct interaction. Alternatively, RIs may collect samples under predefined protocols for users to analyse in their home institutions. *Remote instrument control* adds another flavour, since here the users and not the RI staff remotely operate RI instruments for sample collection or sample analysis via specialised software interfaces ( Figure 1). These modalities, while sharing common challenges, introduce unique complexities to the operation and user engagement strategies of RIs.

When remote access starts with a user-supplied sample, several technical and logistical challenges are involved: sample preparation by the user, intra-/international shipment, and receipt/handling by RI operational staff. For users, this requires knowledge with respect to preparation of samples amenable for destination-specific shipping and regulatory requirements. For RI staff, they should possess a priori knowledge on sample-specific storage, processing, and handling for specific RI instrumentation. Importantly, the latter may require specialised expertise to ensure accurate and efficient handling post-shipment.

The practicalities of sample shipment ^10, 11^ have been a topic consistently discussed in the eRImote Workshops and Expert Groups. Since users and RIs rely on third party courier or shipment services, it is outside their direct control. Delays or adverse shipment conditions may deteriorate samples, compromising measurements to a point where analysis is no longer possible or useful. In addition, jurisdiction-specific regulations and restrictions often apply, which may additionally differ as a function of sample type (e.g. organic versus inorganic samples). During the pandemic, the ban of international travel and resulting grounding of planes also adversely affected international shipments. Political dynamics such as Brexit can dramatically change the regulatory frameworks for sample shipments. Finally, shipment challenges generally increase significantly when samples are sent between continents, increasing barriers to access to RIs for researchers outside Europe.

In this context eRImote experts advocate for the establishment of a unified European regulatory framework for the shipment of scientific samples. This initiative would help to overcome the sometimes-incongruent national patchwork of regulations, increase efficiency by decreasingly regulatory complexity, and improve the leverage of RIs in their negotiations with shipment providers. This recommendation also highlights the need for international collaboration, suggesting that governmental working groups could play a pivotal role in simplifying customs procedures for the international exchange of scientific samples and research purposes, thereby facilitating global research collaborations, while at the same time ensuring (and even increasing) safety ^12^.

Sample handling by the RI involves additional operational resources and qualified staffing level, compared to physical access. This is particularly true if the RI also carries out the entire measurement and analysis process on behalf of the user. Since RI and users need to agree in advance on the nature and scope of the sample characterisation, there is either an increased need for user communication, or the operational processes required need to be highly standardised. While the latter does not exclude the provision of more complex services by any means, it does require a high degree of preparatory work on the part of the RIs and the user. Moreover, during remote instrument control, RI staff must be available for technical support, troubleshooting and to perform tasks that cannot be managed remotely (such as sample placement) further escalating staff resource needs.

Many remote access processes developed by the RIs during the pandemic also have proven useful in combination with the preparation and post-processing of in-person access. This has given rise to an increase in *hybrid access*, where for example initial experiments are carried out by users on-site, while more extensive follow-up experiments are carried out remotely by the RI operational staff. These hybrid models combine remote and physical access, enhancing the versatility of service delivery and potentially enriching the user experience.

*Remote instrument control* necessitates stringent security measures, including robust user authentication and authorization protocols, to protect critical RI equipment from external threats. This involves a comprehensive review of the facility's security architecture. Users must be accurately authenticated to confirm their identity and authorized specifically for instruments they can access. Although RIs may be able to source Authentication and Authorization Infrastructure (AAI) solutions externally, e.g. as part of the European Open Science Cloud (EOSC) architecture, these solutions still require RI-specific technological implementations, such as effective user management systems. Additionally, since many of these solutions depend on external authentication sources like university accounts, alternative authentication methods must be developed for users outside academia or international researchers to ensure broad accessibility.

Remote instrument control relies on software to virtualise instruments and provide user interfaces. In general, when instruments are fully controllable by software on-site, this situation can also be replicated offsite, although it requires adequate IT and network infrastructure at the user end to ensure sufficient resolution and speed. Instruments requiring physical interaction may necessitate modifications for full remote operability, impacting operator training requirements and necessitating additional input from instrument manufacturers to adapt their products for remote use and prevent instrument damage from accidental remote misuse.

While a comparison of different software solutions for remote instrument control is not within the scope of this paper, key considerations include usability, cost and licensing models, and their ability to integrate the existing AAI solutions of the RI. As compatibility with proprietary software from instrument vendors must also be considered, these should be included in discussions with the RIs and the users about the technical, legal, and licensing implications in the context of remote user access to their instruments.

The transition to remote access introduces significant changes in user relationships with Research Infrastructures (RIs) and the data they generate. Remote access increases the distance – both physically and to a certain extend mentally – between the user and the RI, potentially transforming RI staff roles from collaborators and trainers to service providers. The users’ identification with the RI that they use is also likely reduced, with unclear impact on future RI engagement. This may affect the users’ understanding and relationship to the data that is gathered during the remote RI access, raising questions around not just data ownership, legal and ethical issues and intellectual property (IP), but also efficiency and data sharing/reuse. These challenges underscore the specific and increased need to communication between the user and RI staff in the context of remote access, including to clarify concerns around IP, data accessibility and ownership, data FAIRification, and authorship or acknowledgment of RI facility staff in publications.

**Summary Box. Remote Access**

**Identified issues:**

- **Increased staff effort and expertise:** Necessary for sample handling, executing measurements, and providing technical support during remote sessions.

- **Higher up-front efforts by RIs**: Essential for developing remote protocols and enhancing user communication.

- **Higher efforts for RI users:** Users must invest significant effort in preparing samples for shipment and handling by RIs.

- **Logistical and regulatory challenges with sample shipment:** Shipping introduces complexities and potential delays.

- **Enhanced cyber-security measures:** Increased demands for robust user authentication and authorization to safeguard access.

- **Instrumentation access requirements:** Need for intuitive user interfaces and virtualization solutions, potentially impacting licensing models.

- **Elevated training needs:** Both users and RI operators require more training to adapt to remote access technologies.

- **Networking and collaboration efforts:** Additional effort is required to mimic the collaborative environment of physical RI access.

**Recommendations for RI management, funders, and policy makers:**

- **Upgrade IT infrastructure and leverage cloud-based solutions:** Invest in robust and scalable IT infrastructure to support high-quality remote and virtual access, including high-speed internet, secure servers, and advanced software tools. Cloud-based solutions for data storage and processing enhance accessibility and collaboration across different geographical locations.

- **Acknowledge the efforts of research data management:** Setting up appropriate research data management involves procedure definitions, IT infrastructure operation, personnel sustainability and physical infrastructures usage and requires interactions between scientists, IT experts, data managers, lawyers and other key professionals.

- **Acknowledge increased operational efforts:** Recognize that remote access demands more significant operational efforts from RIs compared to traditional physical access. This should be factored into managerial and funding strategies at institutional, national, and European levels.

- **Establish a unified regulatory framework for shipping scientific samples:** Advocate for a cohesive European regulatory framework for the shipment of scientific samples, engaging intergovernmental groups to ease global sample transportation.

- **Adopt best practices for remote instrument control:** Encourage the development and adoption of best practices tailored to the specific technological needs and requirements of RIs for remote instrument control.

- **Enhance training programs:** Implement comprehensive training programs for both RI staff and users to navigate the complexities of remote access and ensure the effective use of remote instruments.

- **Invest in cybersecurity**: Prioritize investments in cybersecurity measures to protect sensitive data and infrastructure, ensuring secure and reliable remote access.

- **Facilitate collaboration and networking:** Develop platforms and tools that foster collaboration and networking among remote users, aiming to replicate the interactive and communal aspects of physical RIs.

- **Monitor and evaluate remote access impact:** Continuously monitor and evaluate the impact of remote access on research quality and user engagement, using these insights to inform future enhancements.

## Virtual access

**Figure 2.**
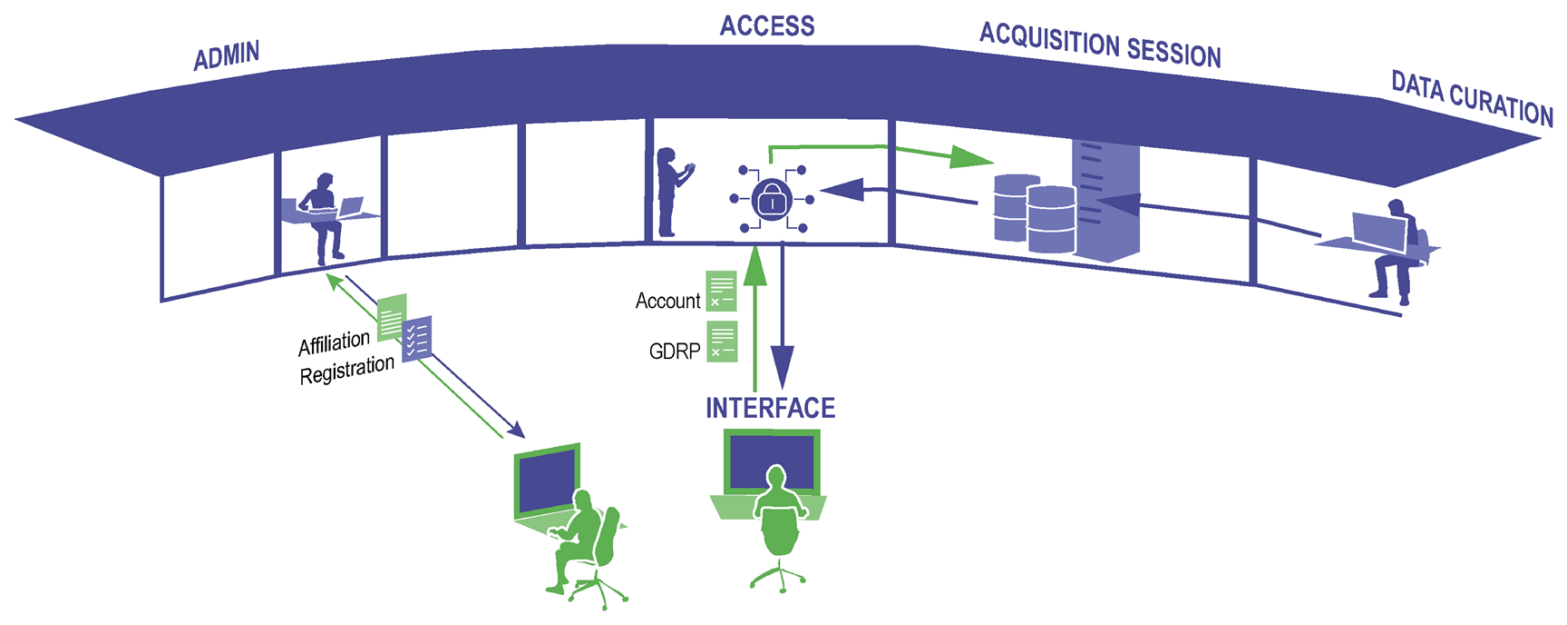
Virtual Access: interaction between the user (depicted in green) and the RI staff (blue). Example of typical elements of virtual access e.g. to data bases, starting from the registration of the researcher, which may require complex verification of ID and affiliation especially in case of access to sensitive data, and moving to data acquisition and curation of the data to which the user obtains access.

Virtual access ( Figure 2), a common access model in the Social Sciences and Humanities RIs, but also applied in many other RI domains, shares characteristics of remote access and may face similar challenges, but often at a higher scale: the standardisation of data management plans and metadata standards across different facilities in distributed RIs, data FAIRification (i.e. making data findable, accessible, interoperable, reusable), high demands on cyber-security, and the sharing of large data volumes, which requires robust high-bandwidth connections and/or temporary data storage solutions, or in other cases the legal restrictions on data movements for sensitive data.

In contrast to physical and remote access, virtual access does not necessarily involve a selection of projects or users. Subject to legal constraints (see below), data objects can be downloaded freely and as often as required. Tracking and accounting for the usage of provided resources is a common challenge in RIs, but this particular model of virtual access where data resources are openly and widely shared, amplifies this issue. Internet protocol (IP) tracking, persistent identifiers for data objects, and mandatory user accounts may provide partial solutions, but they need to be adopted in agreement with the respective user communities. In the context of software development for virtual infrastructures, the source of the funding and sustainability models are unclear, posing obstacles to innovation.

Data provided through virtual access is usually categorised by its granularity, whether it allows identification of personal data, or whether intellectual property (IP) rights and proprietary data resources are linked to the data set. Higher levels of personal or proprietary data in a dataset typically necessitates tighter access controls and regulations. RIs need to provide the means for user access in accordance with these regulations and they need to safeguard the data against misuse, theft, or unauthorised access ^13^.

Legal frameworks such as the General Data Protection Regulation and national regulations on the sharing of health-related data may significantly affect cross-border data access and sharing. The concerted action of national funders and the European Commission to fight the Covid-19 pandemic triggered a surge in collaborative research projects, which often faced significant challenges brought about by a fragmented regulatory environment for data sharing ^14^. In the context of providing sensitive data to the EOSC, communities have developed specifications and frameworks to address the challenges associated with data sharing and access, particularly regarding ethical and legal considerations ^15, 16^.

**Summary Box. Virtual access**

**Identified issues:**

- **Standardization needs:** There's a pressing need for uniform data management plans and metadata protocols across distributed facilities to ensure consistency and interoperability.

- **Usage tracking difficulties:** Tracking the usage of digital objects presents significant challenges, complicating the assessment of data impact and utility.

- **Regulatory constraints:** National and European regulations on personal and sensitive data sharing pose obstacles to cross-border data access, requiring careful navigation.

- **Cybersecurity requirements:** The safeguarding of sensitive data necessitates advanced cyber-security measures, alongside rigorous user authentication and authorization protocols.

**Recommendations for RI management, funders, and policy makers:**

- **Standardise data management and metadata:** Implement uniform Data Management Plan (DMP) templates and metadata standards enhancing machine readability to support automated processing and integration across diverse RIs.

- **Enhance digital object usage tracking:** Advocate for the adoption of Persistent Identifiers (PIDs) at all levels, utilizing analytics tools and software that seamlessly integrate with current databases and platforms, thus ensuring data providers receive due credit and feedback for their contributions.

- **Tackle regulatory challenges in data sharing:** Develop strategies to navigate the complex landscape of national and European regulations affecting cross-border data sharing, facilitating easier access while ensuring compliance.

- **Enhance cybersecurity and data access controls:** Strengthen cybersecurity measures and refine data access protocols to protect sensitive information and maintain data integrity.

- **Expand capacity and awareness:** Launch initiatives aimed at building capacity and enhancing awareness around data management, cybersecurity, and the ethical implications of data sharing to foster a more informed and secure research environment.

## Remote training of RI users and RI staff

The provision of training for users and staff of RIs is a very important part of RI activities for all access types. RIs often include complex and specialised equipment, software, data, and facilities. Training ensures that RI staff and users know how to operate these resources efficiently and make the best use of them. RI operation may also involve hazardous materials, powerful instruments, or sensitive data, requiring training to ensure that staff and users are aware of the safety protocols, security measures, and ethical considerations involved. It helps to avoid unnecessary errors, reduce downtime, and optimise the use of expensive and valuable equipment. Training provided by RIs improves users’ skills and knowledge and enables them to conduct high-quality experiments and data analysis. Properly trained users are more likely to produce reliable and reproducible results, and training received in the context of RI user access can be often applied in other contexts in the users own research.

**Figure 3.**
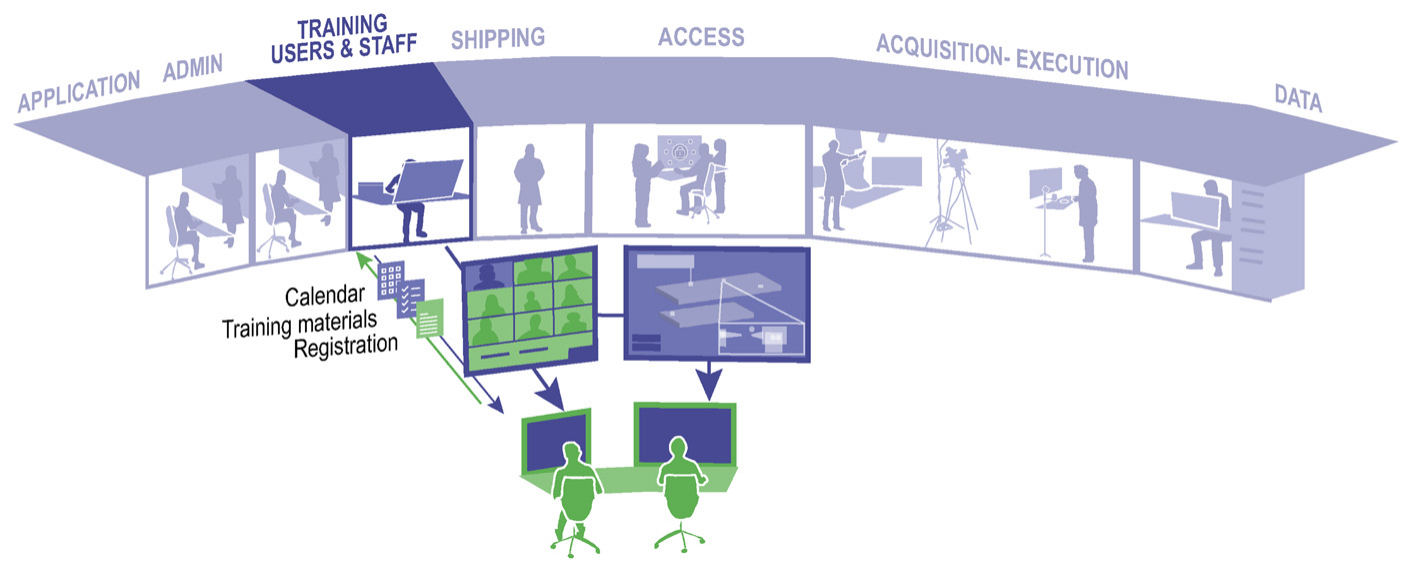
Remote training: interaction between the user (depicted in green) and the RI staff (blue) Example of typical elements of virtual training.

Remote training in RIs ( Figure 3) was already gaining traction prior to the pandemic restrictions, due to its obvious advantages: increased accessibility and flexibility, reach and inclusivity as participants can attend from anywhere, increased cost efficiency as travel and accommodation costs are saved for providers and participants, recording of training sessions allowing asynchronous learning when training material can be reused and revisited for consolidation and future reference, and the resulting consistent training content ensures that all participants receive the same information.

Remote training has, however, also several disadvantages that need to be carefully balanced against the benefits: on-site RI access automatically comes with hands-on experience and training on usage of instruments or technology. Such hands-on training for physical processes is obviously difficult to near impossible to fully replicate remotely. In remote training, opportunities for immediate feedback and spontaneous discussion are limited if not explicitly incorporated into the training programme. In addition, participants may be distracted in their own environment, which affects engagement, and time-zone differences may complicate scheduling.

Remote training generally requires an adapted skill set of the trainers who need to cope with the limitations of indirect interactivity. In addition, training materials need to be adapted to the remote situation and refined and classified for findability. Materials of high granularity require an ontology that provides means of navigation for reuse of the material. Aside from ontologies, data containers including the content and metadata, are required. Access to learning material may require the creation of user accounts and the application of online learning platform solutions. Overall, remote training can be highly effective, but to be so requires a mindset shift to often unfamiliar event programming and teaching methods, as well as a shift in the preparatory burden compared to in-person training, with higher logistics requirements.

The effectiveness of training remotely vs. in-person also depends on the career-stage of the person being trained. Particularly for early career researchers or those new to a particular RI, in-person user training provides a supportive environment to gain hands-on experience and build collaborative networks that are difficult to replicate in remote training. Someone already working with a particular technology or in a certain RI can likely benefit from either in-person or remote learning. This holds true not just for remote training, but also for remote access, which is likely to be more efficient and beneficial for experienced or well-trained users.

Hybrid training solutions may provide some of the advantages of both in-person and remote training, but they may also be significantly more costly than solely in-person or solely online events (up to three times the costs, including staff effort, compared to in-person) and place special requirements on the venues. Therefore, RIs need to consider whether the potentially small increases in attendance are worth the increased investment or how hybrid events can be developed to be equally engaging for online audiences. In addition, considerations on how to produce more long-lasting online training material from hybrid events are needed.

**Summary Box. Remote Training**

**Identified issues:**

- **Enhanced accessibility and flexibility:** Remote and hybrid models significantly expand access and inclusivity but introduce complexities in engagement and experience.

- **Cost efficiency vs. increased costs for hybrid models:** While remote training reduces travel costs, hybrid solutions may inadvertently raise overall expenses.

- **Asynchronous learning opportunities:** Recorded sessions enhance learning flexibility but may diminish the immediacy of interaction.

- **Reduced practical experience:** The lack of hands-on experience in remote settings limits experiential learning.

- **Diminished personal interaction:** Limited face-to-face engagement affects networking and personal connection opportunities.

- **Need for specialised trainer skills:** Adapting to remote formats requires trainers to develop new competencies.

- **Technical requirements for access:** Ensuring seamless access to training materials and platforms is crucial.

**Recommendations for RI management, funders, and policy makers:**

- **Optimise remote training plans:** Incorporate extended breaks, dedicated networking sessions, and interactive tools to mimic in-person engagement.

- **Enhance virtual training materials:** Annotate materials clearly and adhere to FAIR principles for broader accessibility and usability.

- **Strategically plan hybrid trainings:** Address technical needs meticulously and design sessions to benefit both in-person and remote participants effectively.

- **Foster community through digital platforms:** Utilise forums, social media, and interactive platforms to build a sense of community and facilitate networking.

- **Professional development for trainers:** Offer training and resources to help trainers excel in remote and hybrid environments.

- **Evaluate and iterate:** Regularly assess the effectiveness of training methods and materials, incorporating feedback to continuously improve the training experience.

## Further considerations on operational requirements and efficiency of remote and virtual access

As outlined in earlier sections, providing remote and virtual access, along with training, can bring great advantages for both the RIs and their users, but it also significantly increases the operational demands on RIs. This includes an escalation in both efforts required from RI personnel, as well as heightened requirements for operational systems and processes to meet these advanced service expectations.

The drive towards remote and virtual access however also intersects with other trends, such as developments towards automation in large facilities, which make remote access easier. In addition, most systems and processes developed for remote working can also be helpful in a non-remote context, such as allowing RI staff to remotely check in on instruments running 24/7 without having to be at the machine.

Remote and virtual access may pose increased hurdles for RI users, which need to be addressed by clear procedures, communication and user training offers. The absence of direct interactions could shift the dynamic from collaborative research partnerships to a more service-oriented relationship, impacting the collaborative essence of RIs. Proper acknowledgement of RI services in user publications or joint publications could be a partial remedy, but mandatory requirements are difficult to police for the RIs. The lack of direct user interactions may also impact the role Rls play in science diplomacy, where they are places for collaboration, exchange, and for meeting of researchers from many different countries and backgrounds. This often-unseen aspect of in-person Rl usage is important in the current global situation and is lost in remote access.

Discussions within the eRImote project revealed mixed impacts of adopting remote and virtual access on RIs’ operational efficiency. While in some cases RIs reported enhanced efficiency, others saw a decline as compared to the in-person access, underscoring the variable outcomes of transitioning to these access models ^1^. The effects vary, based on the service offer of the RI, how standardised the experimental processes and samples are, and how much user input is needed on experimental fine-tuning. These variations make it difficult to assess the overall impact of remote access on RI operational efficiency without much more detailed exploration than was possible in the context of eRImote.

This is a critical point, since in resource-intensive scientific instrumentation such as neutron sources NMR or MRI machines, or synchrotrons, small decreases in operational efficiency may easily offset any decrease in the carbon-footprint due to reduced travel. The growing energy-consumption impact of large-scale data also has to be taken into account in any considerations of environmental impact of user access. With increasingly large datasets and compute-intense analysis methods (ML/AI), efforts to reduce environmental impact may be more impactfully targetted at questions of reducing data movements while maintaining accessibility and reusing of pre-trained Al models, than the comparatively small environmental impact of travel to RI sites. More work is needed to assess the overall environmental impact of increasing remote access to RI services, also considering the environmental impacts of purchasing and running new equipment when such equipment is already available through existing research infrastructures, even when effective use of such equipment would require physical access.

Such work would ideally aim to pave the way towards a commonly agreed specific methodology to measure the environmental impact of research infrastructures. In order to compare remote to onsite, clarity is needed on whether to consider only the CO _2_-footprint or also other greenhouse gases, but also on which parameters should be used for comparison, the methodology, the considered scope, and assumptions about current and future CO _2_ intensity e.g. of electrical power. The R-NMR project has gathered comprehensive information from the European NMR community, which serves as a highly informative case study on the relative environmental impacts of travel and instrument usage for this type of specialised facilities ^17^.

## eRImote recommendations for facilitating remote and virtual access to research infrastructures, including remote training

1. **Budgeting for and funding of remote access:** Remote access provision usually comes with increased operational costs and training requirements that need to be considered by the RIs themselves when developing their service provision plans, but also by funders when including remote and virtual access into RI access programmes. In particular the national RI funding schemes need to take into account that remote access potentially extends the geographical reach of RIs service provision beyond national borders.
2. **Fostering collaboration and best practises:** There is a continuing need to strengthen collaboration and sharing of best practices among Rls to enhance the efficiency and effectiveness of remote access provision.
3. **Developing a unified regulatory framework:** There is a need for the creation of a unified European regulatory framework for the shipment of scientific samples. Intergovernmental working groups should be involved to simplify sample shipment between continents.
4. **Managing user relationship changes:** RIs need to take into account that remote and virtual access can affect user relationships. Their perception of the RI may shift from scientific collaboration to service provision, with a related change in user expectations and how they value the RI contributions. This can best be mitigated by actively involving RI user communities when implementing remote and virtual access models.
5. **Conducting structured assessments:** Structured assessment of the impact of remote and virtual access on RI operational efficiency and the related environmental consequences are required.
6. **Balancing access models:** Remote and virtual access should not be seen as a 'silver bullet'. There are wide reaching consequences of adopting remote access to RI and the larger the divergence compared to pre-pandemic arrangements, the larger the potential for unexpected and unwanted impacts. This also means that RI policy should not automatically pursue further remote and virtual access in all contexts. A balance between possible RI access options needs to be maintained and each RI can best judge what is the most effective and requested combination of applied access models in their case.
7. **Adapting data sharing policies:** In light of a changing global context, data sharing policies may need to be adapted, emphasising scientific exchanges and collaborations with third countries on the basis of shared values. In this context, research policy needs to provide clear guidelines for the RIs, particularly on the limitations and restrictions imposed by this value-based approach to data sharing.

## Ethics and consent

Ethical approval and consent were not required.

## Data Availability

No data are associated with this article. The views expressed in this article are those of the author(s). Publication in [platform name] does not imply endorsement by [funder name].

## References

[1] European Strategy Forum on Research Infrastructures: ESFRI glossary - Research Infrastructures. Reference Source

[2] eRImote Project Report: Preliminary overview of RI access provision changes during the pandemic. 2023. Reference Source

[3] European Strategy Forum on Research Infrastructures: ESFRI Report on access to research infrastructures and charter on access to RIs. *Zenodo.* 2024. Reference Source

[4] eRImote Consortium: eRImote project website - home. 2022. Reference Source

[5] Galeott M Tegas V : eRImote WP4 D4.2 remote access use case report (1.0). *Zenodo.* 2024. 10.5281/zenodo.14235482

[6] European Commission Directorate-General for Research and Innovation: European charter of access for research infrastructures – principles and guidelines for access and related services.Publications Office. 10.2777/524573

[7] ESFRI: ESFRI white paper - making science happen - a new ambition for research infrastructures in the European research area. 2020. Reference Source

[8] ESFRI Report: Access to research infrastructures and charter on access to RIs. 2023. Reference Source

[9] Cartwright HN Hobson CM Chew TL : The challenges and opportunities of open-access microscopy facilities. *J Microsc.* 2024;294(3):386–396. 10.1111/jmi.13176 36779652

[10] Schweigreiter R Cawthorne C Lippens S : Collaborating by courier, imaging by mail. *EMBO Rep.* 2019;21(1): e49755. 10.15252/embr.201949755 31840417 PMC6945055

[11] Mc Carthy J Chebre A Blasetti C : Report on mail-in sample handling procedures at LEAPS.2022. Reference Source

[12] EU-LAC: Latin America and Caribbean structural biology landscape analysis report. 2022. Reference Source

[13] Lichtwardt B Woollard M Wiltshire D : Setting up a secure remote connection between two trusted research environments.2022. Reference Source

[14] Taconelli E Gorska A Carrara E : Challenges of data sharing in European COVID-19 projects: a learning opportunity for advancing pandemic preparedness and response. *Lancet Reg Health Eur.* 2022;21: 100467. 10.1016/j.lanepe.2022.100467 35942201 PMC9351292

[15] Boiten JW Ohmann C Adeniran A : EOSC-Life guidance and policy on standards and tools to facilitate sharing and reuse of multimodal data (including imaging), cohort integration, and biosamples. 2021. Reference Source

[16] Bishop L Broeder D van den Heuvel H : White paper on remote access to sensitive data in the social sciences and humanities: 2021 and beyond. 2021. 10.5281/zenodo.6719121

[17] R-NMR Consortium: Remote-NMR landscape including table of criticalities. Appendix 4: Monitoring the Carbon Footprint. Reference Source

