# Supplementary material for: Facilitating remote and virtual access provision by European research infrastructures – requirements, issues, and recommendations
Source: Open Res Eur. 2025 May 16;4:152. Originally published 2024 Jul 24. [Version 2] doi: 10.12688/openreseurope.18023.2 (PMC11364971; doi:10.12688/openreseurope.18023.2)
Supplement: Peer review history

# Reviewer report: Reviewer response for version 2

**DOI:** 10.21956/openreseurope.21271.r54286

**Published:** 2025-06-03

**Authors:** Daniele Bailo

**Affiliations:** 1 Istituto Nazionale di Geofisica e Vulcanologia, Roma, Italy; 2 EPOS ERIC, Rome, Italy

Most of the points raised in the review were addressed, so it can be approved

Where applicable, are recommendations and next steps explained clearly for others to follow? (Please consider whether others in the research community would be able to implement guidelines or recommendations and/or constructively engage in the debate)

Yes

Does the article adequately reference differing views and opinions?

Partly

Are all factual statements correct, and are statements and arguments made adequately supported by citations?

Partly

Is the rationale for the Open Letter provided in sufficient detail? (Please consider whether existing challenges in the field are outlined clearly and whether the purpose of the letter is explained)

Yes

Is the Open Letter written in accessible language? (Please consider whether all subject-specific terms, concepts and abbreviations are explained)

Yes

Reviewer Expertise:

I am a researcher at the Istituto Nazionale di Geofisica e Vulcanologia and IT Officer at EPOS ERIC. My work focuses on the integration and innovation of distributed research infrastructures in the Earth sciences, aiming to enhance data accessibility and scientific workflows through advanced digital services. I specialise in designing and implementing data-intensive architectures that enable seamless access to multidisciplinary scientific data, with particular emphasis on interoperability, metadata harmonisation, and the application of machine learning and AI to geoscientific challenges.My scientific interests include the use of artificial intelligence applied to seismology and the exploration of large language models (LLMs) for supporting knowledge extraction and scientific discovery in the geosciences. I am also actively involved in shaping strategy and policy in Open Science and FAIR data, contributing to the governance and technical direction of EPOS ERIC.With a background in computer engineering and extensive experience in European collaborations, I work at the interface between scientific needs and technological solutions. My contributions aim to foster a more connected and intelligent ecosystem for research, enabling cross-domain discovery and innovation through robust and sustainable digital infrastructures.

I confirm that I have read this submission and believe that I have an appropriate level of expertise to confirm that it is of an acceptable scientific standard.

# Reviewer report: Reviewer response for version 1

**DOI:** 10.21956/openreseurope.19478.r42486

**Published:** 2024-08-30

**Authors:** Daniele Bailo

**Affiliations:** 1 Istituto Nazionale di Geofisica e Vulcanologia, Roma, Italy; 2 EPOS ERIC, Rome, Italy

The open letter, in outlining the findings of the eRImote project (European Research Infrastructures - Pathway to Improved Resilience through Digital and Remote Access), aims to identify the benefits, challenges, and barriers for remote and virtual access to Research Infrastructure. It also investigates the remote training benefits and hurdles, and finally provides a set of recommendations for facilitating remote and virtual access to research infrastructures, including remote training.

**Strengths:**

- The reasoning behind the Open Letter is clearly presented, offering a thorough understanding of the current challenges and the letter's intent.
- The letter is written in a clear, understandable and accessible language.
- The scope of the letter is wide, as it aims to address all research infrastructures. Therefore, all RIs can find relevant elements or lessons for the set-up, operation and maintenance of their frameworks (spanning from IT, to governance and sustainability dimensions).
- The key elements of Remote Access, Virtual Access, and remote training are thoroughly described, showing comprehensive analysis of the RIs in the study, making the results valuable for the entire RI community.

**Recommendation for improvement:**

- More examples demonstrating how the issues have occurred in specific RIs and how these have been tackled, would help to make the recommendation more understandable and concrete
- When it comes to Virtual Access, the process of setting up appropriate Research Data Management involving procedure definitions, IT infrastructure operation, personnel sustainability and physical infrastructures usage seems to be a bit underestimated. More emphasis on the considerable efforts to provide and operate a reliable VA is needed, maybe pointing to relevant resources in literature. (e.g., considering the interactions between scientists, IT experts, data managers, lawyers and other key professionals).
- The letter reports that the study is based on 12 partners from diverse disciplines. It would be interesting to know who these partners are, maybe pointing to reports, paper or other material demonstrating how these tackled the issues and recommendations highlighted in the paper. These could be useful lessons to learn from.
- References are limited to 10, but there is significant potential to improve the usefulness of the letter by including more references to the analyzed RIs, use cases, practices, and concepts. In particular, when it comes to recommendations, having pointers to relevant material would help readers from RIs to understand how to implement recommendations.

Where applicable, are recommendations and next steps explained clearly for others to follow? (Please consider whether others in the research community would be able to implement guidelines or recommendations and/or constructively engage in the debate)

Yes

Does the article adequately reference differing views and opinions?

Partly

Are all factual statements correct, and are statements and arguments made adequately supported by citations?

Partly

Is the rationale for the Open Letter provided in sufficient detail? (Please consider whether existing challenges in the field are outlined clearly and whether the purpose of the letter is explained)

Yes

Is the Open Letter written in accessible language? (Please consider whether all subject-specific terms, concepts and abbreviations are explained)

Yes

Reviewer Expertise:

Research Infrastructures, metadata, IT Management, Data science

I confirm that I have read this submission and believe that I have an appropriate level of expertise to confirm that it is of an acceptable scientific standard, however I have significant reservations, as outlined above.

# Reviewer report: Reviewer response for version 1

**DOI:** 10.21956/openreseurope.19478.r42484

**Published:** 2024-08-10

**Authors:** Pasquale Pagano

**Affiliations:** 1 CNR - ISTI, Pisa, Italy

The article thoroughly overviews the need for remote and virtual access to RIs, especially in the COVID-19 pandemic. It effectively outlines the benefits, such as increased accessibility and reduced geographical and financial barriers, as well as the challenges, including regulatory issues and the need for adequate funding.

**Strengths:**

- The rationale for the Open Letter is well-articulated, providing a clear understanding of the existing challenges and the letter's purpose.
- The paper is well-supported by citations, ensuring the accuracy of the information presented.
- The language used is accessible, making the content understandable to a broad audience.
- Recommendations and next steps are clearly outlined, providing actionable guidance for the research community.

The following recommendations could be added to the recommendations for facilitating remote and virtual access to research infrastructures:

- **Upgrading IT infrastructure:** Investing in robust and scalable IT infrastructure to support high-quality remote and virtual access, including high-speed internet, secure servers, and advanced software tools.
- **Cybersecurity measures:** Implementing strong cybersecurity measures to protect against data breaches and cyber-attacks, ensuring the integrity and confidentiality of research data.
- **Cloud-based solutions:** Leveraging cloud-based solutions for data storage and processing to enhance accessibility and collaboration across different geographical locations.

These topics emphasize the importance of having a solid digital foundation to support remote and virtual access, ensuring that the infrastructure can handle the demands of modern research activities.

Where applicable, are recommendations and next steps explained clearly for others to follow? (Please consider whether others in the research community would be able to implement guidelines or recommendations and/or constructively engage in the debate)

Yes

Does the article adequately reference differing views and opinions?

Yes

Are all factual statements correct, and are statements and arguments made adequately supported by citations?

Yes

Is the rationale for the Open Letter provided in sufficient detail? (Please consider whether existing challenges in the field are outlined clearly and whether the purpose of the letter is explained)

Yes

Is the Open Letter written in accessible language? (Please consider whether all subject-specific terms, concepts and abbreviations are explained)

Yes

Reviewer Expertise:

I am a researcher and Technical Director of D4Science at CNR, Italy. My pioneering work focuses on the design and development of distributed virtual research environments (VREs). These environments integrate diverse resources, including physical assets like storage and computing power, as well as digital elements such as software, processes, and data to enable virtual access to digital assets. My research aims to create systems where these resources interact seamlessly, providing valuable functionalities to users. Under my leadership, D4Science has made significant strides in developing infrastructures that enhance scientific collaboration and data sharing across various domains. My expertise in distributed architectures, service-oriented infrastructure, and cloud-oriented architecture has been instrumental in advancing e-Infrastructure enabling middleware. My contributions are pivotal in facilitating more efficient and effective scientific research through innovative technological solutions.

I confirm that I have read this submission and believe that I have an appropriate level of expertise to confirm that it is of an acceptable scientific standard.

# Response

**Published:** 2025-05-08

**Authors:** Michael RAESS

We would like to thank Reviewer 02 for the thorough review of Version 1 of our article. We have aimed to address the comments in Version 2. In particular, we have incorporated the suggested recommendations on upgrading IT infrastructure, using cloud-based solutions, and enhancing cybersecurity measures into the recommendations for virtual access in the respective Summary Box.

# Reviewer report: Reviewer response for version 1

**DOI:** 10.21956/openreseurope.19478.r42479

**Published:** 2024-08-01

**Authors:** Lai Ma

**Affiliations:** 1 University College Dublin, Dublin, Leinster, Ireland

The open letter presents the findings of a study pertaining to remote and virtual access to research infrastructures. The letter format primarily highlights the recommendations and initiate discussions on the issues and challenges addressed. The rationale is clear enough – but may not be for everyone because ‘research infrastructure’ refers to a very wide range of facilities, equipment, platforms, resources, and services. It can mean very many different things for researchers working in different fields. It is ambitious to cover all knowledge fields but the significance is somewhat lost when the concepts and examples are rather abstract. The piece would be more useful if specific, if hypothetical, cases are discussed to illustrate the processes by which remote and virtual access can facilitate research, and the potential challenges. The ambition of a pan-disciplinary approach to address every research area risks talking to no one in particular at all.

 Since the open letter is based on the findings of a project, the data collection and analysis are not included in this piece. This should be clearly stated – and some information about the study should be provided so that the open letter can be read in context. The list of references is rather slim, and some claims can/should be supported by citing relevant sources, for example, regulations regarding the sharing of information, i.e. national security, classification, export controls. There is a substantial body of literature about data repositories and their standards and guidelines.

 Further comments

 1) It is fine to use ‘research infrastructure’ as an abstract term for the open letter, but a more elaborated description should be used in the section ‘remote access’ which seems to be based on certain scenarios that are discipline-specific. Concrete examples are necessary as not every reader is familiar with or has the same imaginary of ‘sample’ and what does remote access actually mean in practice.

 2) Virtual access is considered as ‘a common access model in the Social Sciences and Humanities RIs’ seems to overlook open data has been advocated for every research area. For example, computer scientists share their data and codes on platforms such as GitHub. The shortcoming of the section is again the generality it attempts to achieve, however the abstraction and lack of granularity make the recommendations remotely convincing.

 3)The section ‘Remote training of RI users and RI staff’ discusses ‘trained users’ however it is unclear as to what the term refers to and what the criteria are.

 4) The ownership and management of research infrastructure have not been addressed. It is an important issue as to how the costs of remote and virtual access would be accounted for? How would the access to facilities, databases, and training be granted? In other words, are there potential issues regarding membership and/or the establishment of consortia that may counter the ideal of democratizing science?

Where applicable, are recommendations and next steps explained clearly for others to follow? (Please consider whether others in the research community would be able to implement guidelines or recommendations and/or constructively engage in the debate)

Yes

Does the article adequately reference differing views and opinions?

Partly

Are all factual statements correct, and are statements and arguments made adequately supported by citations?

Partly

Is the rationale for the Open Letter provided in sufficient detail? (Please consider whether existing challenges in the field are outlined clearly and whether the purpose of the letter is explained)

Partly

Is the Open Letter written in accessible language? (Please consider whether all subject-specific terms, concepts and abbreviations are explained)

Partly

Reviewer Expertise:

Meta-research

I confirm that I have read this submission and believe that I have an appropriate level of expertise to confirm that it is of an acceptable scientific standard, however I have significant reservations, as outlined above.
